# Syllabus of Obstetrical Lectures

**Published:** 1893-07

**Authors:** 


SYLLABUS OF OBSTETRICAL LECTURES, by Richard C. Norris, A. M., M. D., Philadelphia. W. B. Saunders, $2.00.

A little book designed solely for the medical student and to take the place of note-taking in the lecture room.

